# Immunological diagnosis as an adjunctive tool for an early diagnosis of tuberculous meningitis of an immune competent child in a low tuberculosis endemic country: a case report

**DOI:** 10.1186/s13104-017-2444-9

**Published:** 2017-03-13

**Authors:** Serena Vita, Camilla Ajassa, Emanuela Caraffa, Miriam Lichtner, Claudia Mascia, Fabio Mengoni, Maria Grazia Paglia, Cristina Mancarella, Davide Colistra, Claudio Di Biasi, Rosa Maria Ciardi, Claudio Maria Mastroianni, Vincenzo Vullo

**Affiliations:** 1 Department of Public Health and Infectious Diseases, Sapienza University, 00185 Rome, Italy; 2 Department of Public Health and Infectious Diseases, Sapienza University, Polo Pontino, 04100 Latina, Italy; 3 Microbiology Laboratory, National Institute for Infectious Diseases, Lazzaro Spallanzani, 00149 Rome, Italy; 4 Department of Neurology and Psychiatry, Division of Neurosurgery, Sapienza, 00185 Rome, Italy; 5 Department of Emergency and Acceptance, Unit of Radiology, Policlinico Umberto I, Rome, Italy

**Keywords:** Tuberculous meningitis, Children, Hydrocephalus, Immunological diagnosis, ICCFC

## Abstract

**Background:**

Pediatric tuberculous meningitis is a highly morbid, often fatal disease. Its prompt diagnosis and treatment saves lives, in fact delays in the initiation of therapy have been associated with high mortality rates.

**Case presentation:**

This is a case of an Italian child who was diagnosed with tuberculous meningitis after a history of a month of headache, fatigue and weight loss. Cerebrospinal fluid analysis revealed a lymphocytic pleocytosis with predominance and decreased glucose concentration. Microscopy and conventional diagnostic tests to identify *Mycobacterium tuberculosis* were negative, while a non classical method based on intracellular cytokine flow cytometry response of CD4 cells in cerebral spinal fluid helped us to address the diagnosis, that was subsequently confirmed by a nested polymerase chain reaction amplifying a 123 base pair fragment of the *M. tuberculosis* DNA.

**Conclusions:**

We diagnosed tuberculous meningitis at an early stage through an innovative immunological approach, supported by a nested polymerase chain reaction for detection of *M. tuberculosis* DNA. An early diagnosis is required in order to promptly initiate a therapy and to increase the patient’s survival.

**Electronic supplementary material:**

The online version of this article (doi:10.1186/s13104-017-2444-9) contains supplementary material, which is available to authorized users.

## Background

Tuberculous meningitis (TBM) represents roughly 1% of all cases of TB and it is associated with high mortality and residual neurologic sequelae, even with adequate treatment. In endemic countries the highest incidence of TBM is reported in children aged 2–4 years [1]. Early diagnosis is notoriously difficult and often delayed and it has long been recognized as the single most important factor determining outcome [2]. In fact the microbiological diagnosis of TBM is difficult due to the paucibacillary nature of the cerebrospinal fluid (CSF) in which the microscopy for acid-fast bacilli (AFB) and culture for *Mycobacterium* (*M.*) *tuberculosis* have a low sensitivity [1, 3, 4]. Also commercial nucleic acid amplification tests (NAATs) showed a low sensitivity and specificity compared to culture for the diagnosis of TBM [5]. An additional test is the Adenosine deaminase (ADA) measurements in CSF [6], even if the specificity is low and the cut-off level has not been determined [7].

We present a case study of an Italian child with TBM who was hospitalized in the Pediatric Infectious diseases department of Umberto I° Hospital in Rome, and in whom the early diagnosis, based on immunological flow cytometry test and molecular assay, allowed us to start an early treatment, fundamental requirement to increase patient’s survival.

## Case report

**Fig. 1:**
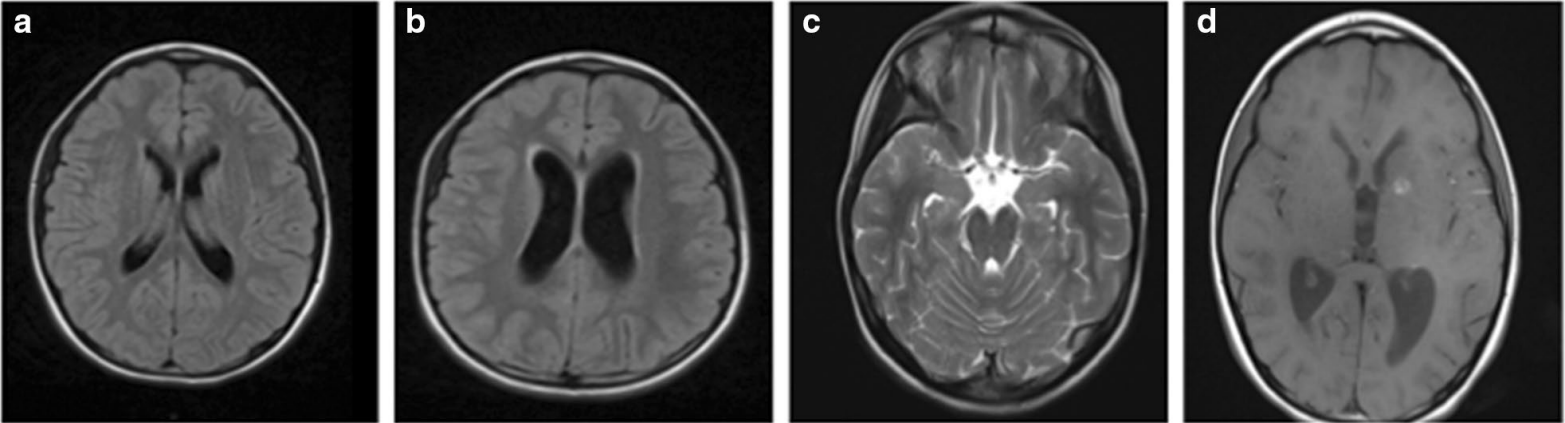
Brain magnetic resonance imaging (MRI). At the onset of the symptoms normal imaging was found (**a**), after 2 days leptomeningeal enhancement over the basilar cistern and hydrocephalus were relieved (**b**, **c**). At admission day 27, focal areas of signal restriction in correspondence of the left caudate nucleus and of the posterior arm of the left internal capsule (**d**) were found suggesting a tuberculous cerebral vasculitis (TVC) as a complication

A 9-year-old Italian girl developed headache, fatigue and weight loss of about 6 kg 1 month prior the admission to our hospital. One week before admission, because of the occurrence of dizziness and vomiting, she was hospitalized to the Neurology Department of another hospital in Rome, where the computed tomography (CT) scan of head and audiovestibular exams were normal. The patient was discharged with a diagnosis of “Symptomatic paroxysmal vertigo, migraine without aura and acute gastroenteritis”. Few days after the discharge, she reported history of sleepiness, persistence of migraine and fever. Thereafter, she was admitted to the Pediatric Emergency Department of our hospital where she had normal blood tests and a normal brain magnetic resonance imaging (MRI). The MRI is shown in Fig. 1a. A normal ocular fundoscopy was performed due to onset of double vision and she was transferred to the Department of Neuropsychiatry where she underwent electroencephalogram (EEG) characterized by slow focal abnormalities. During the hospitalization, the patient experienced increased sleepiness and showed facial nerve palsy with a mouth deviation to the right side for which she was subjected to a lumbar puncture (LP) that showed a clear CSF with a white cell count (WBC) of 372/µL, a lymphocyte percentage of 90%, a protein level of 1.317 mg/dL, a glucose level of 13 mg/dL. Examination of Gram, Ziehl-Neelsen stain and soluble antigen test (*Neisseria meningitis* A-C-Y-W135 Ag, *Streptococcus pneumoniae* Ag) were negative; cultures for common pathogens and *M. tuberculosis,* quantitative polymerase chain reaction (PCR) to detect herpetic viruses (Herpes Simplex Virus-1, 2 and 6, Cytomegalovirus, Epstein Barr and Varicella Zoster Virus) were negative. The GeneXpert MTB/RIF assay (Cepheid) was also negative for CSF. In order to investigate other CNS diseases we performed the Link index [8] and oligoclonal band screen that resulted respectively normal and absent in CSF. Under a suspected diagnosis of clear liquor meningitis, the patient was transferred to our ward. Patient’s past medical history revealed no referred contacts with TB infected subjects. The screening test for human immunodeficiency virus and chest X-ray (CXR) were negative. Tuberculosis Skin Test (TST) was negative and QuantiferonTB-gold in peripheral whole blood (QuantiFERON-TB© Gold In Tube [(QFT-IT); Cellestis Limited Chadstone, Vic., Australia] was indeterminate.

In order to improve the diagnosis of TB, we performed an alternative immunological method based on multifunctional T cells, which has been suggested in recent years as a new tool for the discrimination between active TB and latent TB infection (LTBI) [9]. In a previous study [10] we used an intracellular cytokine flow cytometry (ICCFC) protocol to asses mono-functional and multi-functional *Mtb*-specific CD4+ in peripheral blood and we proposed an immune-based approach, which could improve the identification at single time point of subjects with no TB infection or patients having active or latent TB. A receiver operating characteristic (ROC) analysis was performed to calculate optimal cut-off values for both activated CD4+ T cells and polyfunctional CD4+ T cells in order to discriminate infected (active TB and LTBI) from uninfected patients and active from latent stage. We performed a ROC analysis, and a cut-off >0.45% for activated CD4+ T cells was found as the value allowing the best combination of sensitivity (94.44%, 95% CI 72.2–99.8%) and specificity (100%, 95% CI 69.15–100%; AUC 0.9722; 95% CI 0.9141–1.030%, *P* < 0.0001) to differentiate Mtb-infected patients (active TB and LTBI) from healthy controls [10]. A further cut-off <0.182% for polyfunctional CD4+ T cells allowed the best combination of sensitivity (77.78%) and specificity (70%) to differentiate between active TB and LTBI subjects.

Although this study involved pulmonary TB, we tried to apply our algorithm to TB meningitis. The same method to stain peripheral blood was used to stain CSF. CSF (0.5 mL) was added to the 3 tubes of QFT-IT containing respectively, saline solution (negative control), phytohaemagglutinin (positive control), and TB antigens (ESAT-6, CFP-10, and TB 7.7). A costimulation with 5 μL/mL anti-CD28 plus anti-CD49d (BD Bioscience, Pharmingen, Italy) and 10 μg/mL BrefeldinA (Sigma-Aldrich) was added in all tubes [10]. After 18 h of incubation, the cell surface staining was performed with the markers anti-CD45-VioBlue and anti-CD4 PE-Vio770 (Miltenyi Biotec, Germany), then the cells were lysed (BD Bioscience Lyse solution) and permeabilized (BD Bioscience Perm solution) and the intracellularly stained with anti-IFN-γ FITC, anti-TNF-α APC and anti-IL-2 PE (Miltenyi Biotec). Eventually cells were acquired with a MACSQuant Analyzer flow cytometer (Miltenyi Biotec) and analysed with FlowJo Software version 10, that allowed us to perform a “combination gates” analysis. Seven different population cells were detected in CD4+ cell gate on the basis of IFN-γ, IL-2, and TNF-α produced by CD4+ (Additional file 1), as previously described [10].

**Fig. 2:**
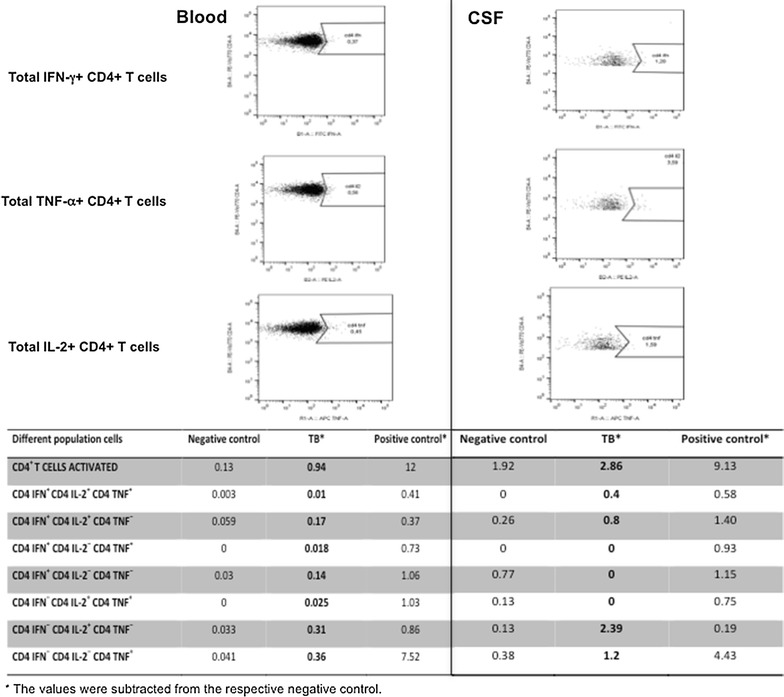
Flowcytometric analysis of CD4+ T cells of blood and CSF after stimulation with TB antigens. The gating strategy excluded debris and identified CD4+ on CD45+ lymphocytes. The subsequent analysis was on CD4+ gate to describe IFN-γ, IL-2, and TNF-α total producing T cells. At the bottom the percentages of the different population cells, calculated by FlowJo Software version 7.6.5 were showed and were defined in CD4+ cell gate on the basis of total IFN-γ, IL-2, and TNF-α producing. T cells producing any of the 3 cytokines (IFN-γ or IL-2 or TNF-α) were defined as “activated T cells”

The percentage of CD4+ T cells activated, defined as cells producing at least a cytokine, was elevated in peripheral whole blood (WB) (0.94%) and in CSF (2.86%) and using the cut-off (>0.45%) we scored the patient as a positive subject TB infected. Worthy of note, at this time the TST was negative, Quantiferon TB-gold was undetermined and a second PCR for *M. tuberculosis* in CSF was negative. Considering the polyfunctionality of T CD4+ cells, those simultaneously producing IFN-γ+ , IL-2+ and TNF-α+ in WB were 0.01% and in CSF 0.40% of total CD4+ cells (Additional file 2). According to our cut-off (<0.182) the patient resulted as active TB in WB assay. Conversely in CSF a higher proportion of polyfunctional CD4+ (Fig. 2) was found comparable to the proportion registered in WB in LTBI patients. To date, few data are available regarding the measure of ICCFC at the site of TB infection, but it could be related to a high immunological response in the CSF, typical of TBM.

Moreover we carried out on the CSF extracted from the LP in the first day of hospitalization, the l ADA level which amounted to 18.31 UI/L, higher than the cut-off 10 [11].

Two days later, due to persistent clinical symptoms and uncertainty in the tests of coordination, a second brain MRI was performed with a report of hypertensive hydrocephalus and a diffuse leptomeningeal enhancement of the basal cistern, in particular of the interpeduncular cistern, attributable to infectious-inflammatory alterations. The brain MRI is shown in Fig. 1b, c. An encephalic drainage was inserted urgently.

Based on the clinical, radiological findings and CSF results (CD4+ polyfunctional response, ADA level, pleocytosis, hyperproteinorrachia and hypoglicorrachia) TBM was considered on admission day 3 and therapy was started with antituberculous drugs with isoniazid (H) 300 mg/day plus ethambutol (E) 500 mg/day plus rifampicin (R) 400 mg/day and pyrazinamide (Z) 1000 mg/day, as well as intravenous steroids (dexamethasone 4 mg every 8 h).

Analysis of CSF obtained from LP and encephalic drainage in the following days revealed a different protein concentration, with higher level of protein in CSF extracted from LP compared to CSF extracted by ventricular drain (Table 1).

**Table 1:** Serial CSF data from lumbar puncture and ventricular drain

CSF data	Day 1 CSF-LP	Day 9 CSF-LP	Day 14 CSF- LP	Day 18 CSF- LP	Day 18 CSF-VD	Day 21 CSF-LP	Day 21 CSF-VD	Day 27 CSF-LP	Day 27 CSF-VD	Day 40 CSF-LP	Day 52 CSF-LP
Appearance	Clear	Clear	Clear	Clear	Clear	Clear	Clear	Clear	Clear	Clear	Clear
WBC count/µL	372	919	321	139	62	718	21	628	120	1.025	291
Neutrophil %	4%	1.3%	9%	17%		11%		6%		4%	20%
Lymphocyte %	90%	98%	81%	74%	61%	78%	78%	90%	84%	91%	72%
Glucose mg/dL	13	15	13	16	45	16	58	9	41	18	18
Protein level mg/dL	1.317	1.993	3.367	2.083	801	14.444	1.746	>6.000	1.881	2.097	2.512
Lactic acid mg/dL	64.9		65.8	56,8	32.4	56.8	25.2	45.1	45.1	44.1	36.9
ADA (U/L)	18.31		9.18	5.02				10.24		4.05	2.89
Direct gram stain	Neg	Neg	Neg	Neg	Neg	Neg	Neg	Neg	Neg	Neg	Neg
PCR *M. tuberculosis*	Neg	*Pos* ^a^									Neg
Culture *M. tuberculosis*	Neg	Neg	Neg	Neg	Neg	Neg	Neg	Neg	Neg	Neg	Neg
Bacterioscopic exam for AFB	Neg	Neg	Neg	Neg	Neg	Neg	Neg	Neg	Neg	Neg	Neg

*LP* lumbar puncture, *VD* ventricular drain, *AFB* acid-fast bacilli

^a^In house nested PCR, while the GeneXpert MTB/RIF assay was negative

Renal and liver function, bilirubin and uric acid levels were monitored regularly. Serial CSF values are shown in Table 1. On day 9 a “in house” nested PCR amplifying a 123 base pair fragment of the *M. tuberculosis* DNA was performed using CSF with a positive result, while the The GeneXpert MTB/RIF assay was negative.

The family screening was performed and only the father was found TST positive with normal chest radiography and chest CT scan.

Once the patient’s condition stabilized, the HERZ regimen was continued and dexamethasone was gradually decreased. However, on admission day 27, because of an acute onset of aphasia with right hemiplegia, an emergency brain MRI was performed (Fig. 1d). The findings were compatible with tuberculous cerebral vasculitis (TVC), resulting in a diagnosis of ischemia with two focal areas of signal restriction in correspondence of the left caudate nucleus and of the posterior arm of the left internal capsule. We increased the corticosteroid dosage and treatment with enoxaparin and aspirin was added.

On admission day 74, there were no further episodes of fever, the neurological examination was normal with total disappearance of hemiplegia, the facial asymmetry and the double vision, thanks to neurological rehabilitation. The patient was discharged in good condition without significant neurological sequelae besides the presence of a slight disorder of attention and concentration and with recommendation to continue the treatment with HERZ regimen and aspirin. She was followed up at the outpatient clinic and a follow-up MRI of brain is shown on the Fig. 1d. Child is on regular follow-up.

## Discussion and conclusions

We report a case of TBM complicated with hydrocephalus and cerebral vasculitis tuberculosis (TVC), which benefited from early diagnosis, based on unconventional methods, and an early TB treatment increasing the chance of a favourable outcome.

TBM remains the most lethal form of *M. tuberculosis* and a recent large childhood study documented neurological disability in about 75% of survivors, despite an exceptionally low mortality rate of only 13% and a treatment with antituberculosis chemotherapy [12].

Early diagnosis and treatment of TBM is the single most important factor determining outcome [13–15]. Although prompt and rapid identification of TBM is crucial for a successful disease management, in most cases, the diagnosis is significantly delayed with a consequent delay in the initiation of therapy, which is often attributable to the use of slow or relatively insensitive conventional diagnostic tests [16].

The diagnosis of TBM is difficult because of unspecific symptoms and signs.

There are some evidences that a combination of clinical and simple laboratory data might help in the diagnosis [17]. Certain clinical characteristics such as longer duration of symptoms (>6 days), moderate CSF pleocytosis, and the presence of focal deficits increase the probability of TBM [17–19]. Characteristic CSF findings of TBM include lymphocytic-predominant pleocytosis, a total white cell count of 100–500 cells/μL, elevated protein levels (typically between 100 and 500 mg/dL) and low glucose, usually less than 45 mg/dL or CSF: plasma ratio <0.5 [20]. Marais et al. [19] have created a score based on clinical, CSF data and cerebral imaging criteria plus evidence of TB elsewhere. According to these authors, probable TBM is defined by a score between 10 and 12, whereas possible TBM is defined by a score higher than 6. At the admission to our ward the patient obtained a Marais score of 6 (4 scores for the CSF findings and 2 scores for clinical criteria) that was insufficient to speculate a TBM diagnosis.

In our case report neither the microscopy, to detect acid-fast bacilli in the CSF, nor culture resulted positive since TBM is a paucibacillary form of tuberculosis so they were unhelpful in making diagnosis. The likelihood of seeing or culturing *M. tuberculosis* from the CSF is dependent upon meticulous microscopy and culture of a large volume (>5 mL) of CSF [21] that is difficult in most cases, especially in children. In fact microscopy, although rapid and inexpensive, has very low sensitivity (10–20%), while culture is too slow and insensitive (<50%) to aid clinical decision-making [17].

In the last decades nucleic acid-based amplification (NAA) tests have emerged as potentially important tools for diagnosing TBM, unfortunately they have a high specificity (97–99%) but low sensitivity (46–66%) [22]. In our patient the first molecular test used (GeneXpert MTB/RIF assay, Cepheid) was negative. Improving the conditions of the CSF sample pre-treatment prior to DNA extraction and increasing the sensitivity of molecular test using a nested PCR [23], it was possible to confirm the suspect of TBM in our patient.

To increase the chance to detect TB infection we used whole blood QFT-G-IT (an Interferon Gamma Release Assay, IGRA) to detect the response to stimuli with specific TB antigens. Our child obtained an IGRA on whole blood indeterminate, in accordance with Vidhate and colleagues [24] who found that whole blood QFT-G-IT had low sensitivity and specificity in diagnosing TBM and CSF QFT-G-IT was useful neither in diagnosis nor in predicting the outcome, due to indeterminate results.

**Fig. 3:**
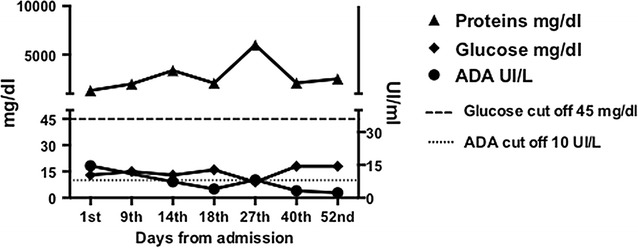
Trend of proteins, glucose and ADA levels in CSF at the different time points. The protein level was always high, while the glucose level was constantly low. The ADA level showed a slow decline after the beginning of therapy

We used unconventional diagnostic method to address the diagnosis. We measured the adenosine deaminase activity (ADA), an enzyme that is widely distributed in tissues and body fluids, that has been used in the diagnosis of pleural, meningeal and pericardial TB [25]. Various studies have demonstrated that CSF-ADA estimation can differentiate TBM from normal subjects or other infectious meningitis [26, 27]. We measured ADA level by a spectrophotometric method described by Guisti and Galanti [28] and it resulted positive only in the first determination at day 1 (Fig. 3). Once we started the anti-TB treatment, it always resulted normal except on day 27 when the child had a TVC and the level slightly increased. ADA measurement on CSF has contributed to diagnosis although the cut-off level that defines a positive result has not been determined [7] limiting the use of this test.

Furthermore we performed an innovative assay to detect the CD4 intracellular response in whole blood and CSF based on the premise that mononuclear cells localised to infected sites produce more cytokines than peripheral blood mononuclear cells (PBMC) [29], which has also been demonstrated in other tissues [30, 31]. Intracellular response in peripheral blood showed firstly a TB infection, revealed by neither TST nor Quantiferon. Moreover the polyfunctional CD4 profile suggested a pattern associated to active TB, according to the previously proposed algorithm [10]. The same test performed in CSF confirmed a *M. tuberculosis* infection with a high local immune specificity. In CSF the increased proportion of CD4 cells producing cytokines (IL-2, INF-γ, TNF-α) could reflect the high number of mononuclear cells localised to infected sites, typical of TBM. A similar phenomenon was described in pleural [30] and alveolar fluid [31] in thoracic TB.

In our study the use of non conventional methods to detect TBM, such as intracellular staining of peripheral blood and of CSF followed by the detection of ADA activity and a in house nested PCR, helped us in the early diagnosis and treatment of TBM, despite the consecutive negativity of classical diagnostic tests. A prospective study with a large number of children is needed to confirm this isolate observation.

## Additional files

**Additional file 1.** Flow cytometry gating strategy for determination of CD4+ T cells responding to TB antigens in blood. Whole blood was analysed using a gating strategy to exclude debris and to identify CD4+ T cells on CD45+ lymphocytes. The subsequent analysis was on CD4+ cells to identify IFN-*γ*, IL-2, and TNF-α production in response to saline solution (negative control), TB antigens and phytohaemagglutinin (positive control).

**Additional file 2.** Flow cytometry gating strategy for determination of CD4+ T cells responding to TB antigens in CSF. CSF was analysed using a gating strategy to exclude debris and to identify CD4+ T cells on CD45+ lymphocytes. The subsequent analysis was on CD4+ to identify IFN-*γ*, IL-2, and TNF-α production in response to saline solution (negative control), TB antigens and phytohaemagglutinin (positive control).

## Abbreviations

TBM: tuberculous meningitis
CSF: cerebral spinal fluid
ICCFC: intracellular cytokine flow cytometry
NAA: nucleic acid-based amplification
ADA: adenosine deaminase
