# Supplementary figures and images for: Immunological diagnosis as an adjunctive tool for an early diagnosis of tuberculous meningitis of an immune competent child in a low tuberculosis endemic country: a case report
Source: BMC Res Notes. 2017 Mar 13;10:123. doi: 10.1186/s13104-017-2444-9 (PMC5347815; doi:10.1186/s13104-017-2444-9)

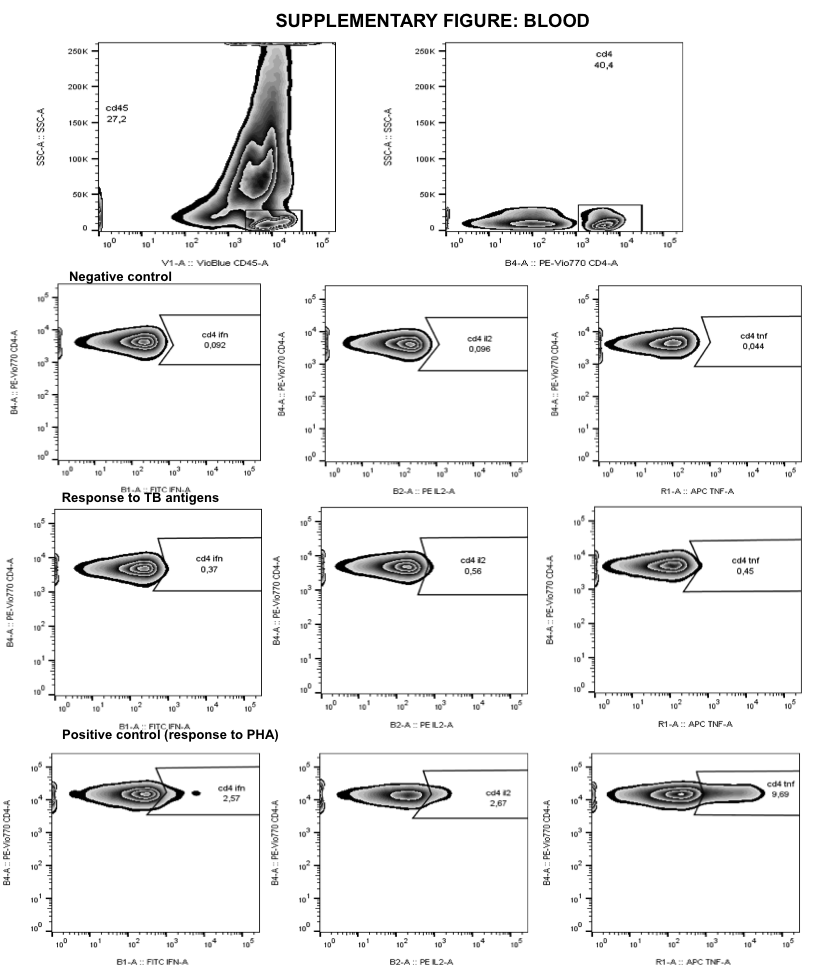

Supplement: Supplementary file 1 — Additional file 1. Flow cytometry gating strategy for determination of CD4+ T cells responding to TB antigens in blood. Whole blood was analysed using a gating strategy to exclude debris and to identify CD4+ T cells on CD45+ lymphocytes. The subsequent analysis was on CD4+ cells to identify IFN-γ, IL-2, and TNF-α production in response to saline solution (negative control), TB antigens and phytohaemagglutinin (positive control). [file 13104_2017_2444_MOESM1_ESM.tif]

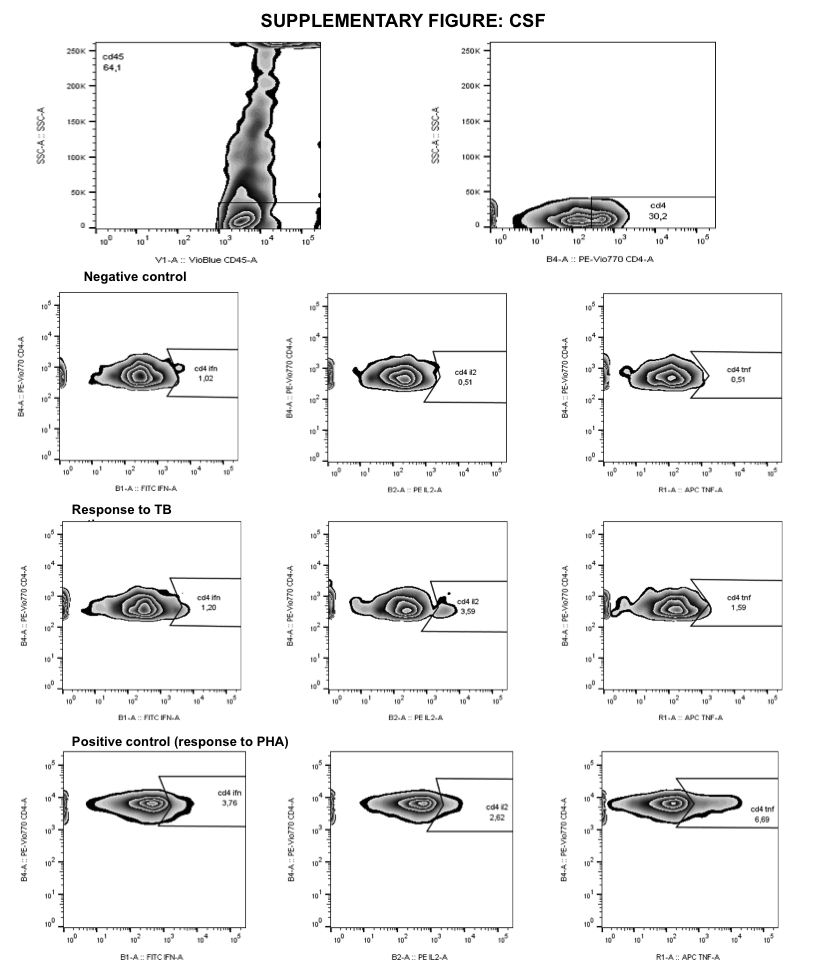

Supplement: Supplementary file 2 — Additional file 2. Flow cytometry gating strategy for determination of CD4+ T cells responding to TB antigens in CSF. CSF was analysed using a gating strategy to exclude debris and to identify CD4+ T cells on CD45+ lymphocytes. The subsequent analysis was on CD4+ to identify IFN-γ, IL-2, and TNF-α production in response to saline solution (negative control), TB antigens and phytohaemagglutinin (positive control). [file 13104_2017_2444_MOESM2_ESM.tif]
